# The (Un)Willingness to Keep Living at Very Advanced Ages

**DOI:** 10.1093/geroni/igab046.3443

**Published:** 2021-12-17

**Authors:** Oscar Ribeiro, Lia Araujo, Laetitia Teixeira, Rosa Marina Afonso

**Affiliations:** 1 University of Aveiro, University of Aveiro, Aveiro, Portugal; 2 Polytechnic Institute of Viseu, Viseu, Viseu, Portugal; 3 ICBAS - University of Porto, ICBAS.UP, Porto, Portugal; 4 University of Beira Interior, University of Beira Interior, Castelo Branco, Portugal

## Abstract

A long life is a general desire that will be reached by more and more people, particularly in developed countries. But the delay of mortality raises important questions about quality of life in the later years. Centenarians have received attention from different disciplines, particularly from demography and genetics, but a psychological approach on whether life at age 100 is perceived as worth living is still very limited. This study explores centenarians’ will to live and associated factors in a sample of 121 centenarians (mean age 101 years; SD 1.63 years; 84.3% female), who answered to a questionnaire comprising sociodemographic characteristics, health status, social functioning, and well-being as well as open questions on their will to live and end-of-life issues. Of the total sample, 31.4% expressed willingness to live longer, 30.6% did not, and 38% presented no clear positioning. From the qualitative thematic analysis, annoyance, uselessness, loss of meaning, disconnection, and loneliness were the most common justifications for being reluctant to live longer. Positive valuation of life and good self-rated health, followed by having a confidant and reduced pain frequency, were the reasons for being willing to live longer. From the quantitative analysis, associated factors of will to live include pain frequency, self-rated health, having a friend confidant and valuation of life. This study provides researchers suggestions for further investigation and highlight the importance of inquiring and understanding very old people’s values and views on their will to live, future wishes, and meaning in life.

